# Agar Plate Methods for Assessing the Antibacterial Activity of Thyme and Oregano Essential Oils against *S. epidermidis* and *E. coli*

**DOI:** 10.3390/antibiotics11121809

**Published:** 2022-12-13

**Authors:** Chiara Mollea, Francesca Bosco, Davide Fissore

**Affiliations:** Department of Applied Science and Technology, Politecnico di Torino, 10129 Turin, Italy

**Keywords:** *Thymus vulgaris* EO, *Origanum vulgaris* EO, *S. epidermidis*, *E. coli*, agar disk diffusion test, disk volatilization test

## Abstract

The resistance to antimicrobials (AMR), especially antibiotics, represents a serious problem and, at the same time, a challenge. In the last decade, a growing interest in the use of essential oils (EOs) as antimicrobial substances was observed. Commercial thyme and oregano EOs are reported to be the main responsible of the oil antimicrobial efficacy against both Gram-positive and Gram-negative pathogenic bacteria. The aim of the present work was to study the efficacy of EOs against *Staphylococcus epidermidis* and *Escherichia coli* in long-time treatments. In a preliminary microdilution test, a MIC value was obtained for thyme EO against *S. epidermidis* and *E. coli*. After that, disk diffusion and disk volatilization tests were set up to study the influence of main cultural parameters on EO activity in liquid or vapor phase. Both bacteria were inhibited by thyme and oregano EOs when applied pure (100% *v*/*v*) or diluted (75% and 50% *v*/*v*): a higher inhibition was observed in a disk diffusion test in which the antimicrobial effect was due to both liquid and vapor phase components. Finally, a comparison with literature data was carried out even if it was not so easy because standard methods are usually modified and adapted to specific case study. For this reason, the results have to be interpreted in relation to the analytical method applied.

## 1. Introduction

The problem of resistance to antimicrobials (AMR) necessitates the identification of new, safe, and effective substances. In recent years, particular attention was paid to products of natural origin due to their low toxicity, biodegradability, and their broad spectrum of action compared to synthetic antimicrobial substances [1]. In this regard, a growing interest in the use of essential oils (OE) was observed since they have two interesting properties: the synergy of action with antibiotics and the absence of acquisition of microbial resistance to their constituents [1].

Essential oils (EOs), secondary metabolites of vegetable origin, are known to be blends of various polar and non-polar chemical molecules such as terpenes, terpenoids, and phenolics [2]. Thanks to this chemical composition, EOs have been studied for many years for their peculiar antimicrobial, antiviral, and antioxidant properties. On the other hand, they also possess some characteristics that can pose some concerns when used: in fact, EOs are volatile, scarcely water soluble (they can be dissolved only in alcohols or organic solvents), and often are characterized by a distinguishing aroma due to volatile components [3]. Nevertheless, thanks to their predominant biological activities, EOs can be utilized in very different fields, from healthcare to the field of food preservation but also in cosmetic product formulations or as repellents in the agricultural environment [4]. EOs can be divided into two main chemical groups: the one of terpenes and that of the aromatic and aliphatic molecules, which includes alcohols, aldehydes, phenols, heterocycles, and methoxy derivatives [5]. Among this last, carvacrol and thymol are reported to be the main ones responsible for the oils’ antimicrobial efficacy. Thyme and oregano, containing these two phenolic compounds, are reported in the scientific literature for their antimicrobial activity against both Gram-positive and Gram-negative pathogenic bacteria such as *Escherichia coli*, *Staphylococcus aureus*, and *Staphylococcus epidermidis* and are mainly tested with agar diffusion assays [6,7]. As an example, in [6], 10 µL of thyme EO obtained similar results against both *E. coli* and *S. aureus* (inhibition halo greater than 20 mm); in [7], *S. aureus* and *S. epidermidis* proved to be resistant to the same volume of thyme EO.

Thyme EO was described as a natural preservative with antimicrobial properties more evident against Gram-positive bacteria probably due to the absence of the external membrane and a cell wall less resistant to the activity of EOs and their components [8]. It contains both thymol and carvacrol in different quantities depending on the plant origin: the antimicrobial activity is mainly ascribed to thymol, but both compounds can have antagonistic or synergistic effects depending on the temperature, the oxygen content, and the pH values [9]. Thanks to its broad spectrum of action, thyme EO was studied for the potential antibacterial activity against food pathogens: in fact, it was added to the formulation of meat, fish, and sea-food in the range 0.1–1%. Moreover, numerous medical applications consider the utilization of thyme EO for its anti-inflammatory, antitussive, analgesic, sedative, and anti-broncholitic properties [8,10].

As regards the antimicrobial efficacy of oregano EO, it mainly depends on the presence of carvacrol, which can interact with the other oregano constituents to enhance efficacy [11]. As for the Thyme EO, its applications are numerous, with oregano being utilized as a food preservative (e.g., in food packaging) or for the treatment of numerous diseases and infections [12].

In a recent review of [13], methods for in vitro evaluation of antimicrobial activity were reported and compared. The authors clearly identified the most important factors that influence the minimum inhibitory concentration (MIC): incubation conditions, culture media, and the use of emulsifiers or solvents. Other factors, such as inoculum size and end-points determination, were mentioned by [14]. In the work of [15], the influence of the most common media used for MIC determination of oregano EO by broth dilution method were reported.

Agar methods for the in vitro evaluation of the EO antimicrobial activity are reported in [14]; among them, the most known is the disk-diffusion assay, based on the Kirby–Bauer standard method, which allows to evaluate antimicrobial activity of the non-volatile components of the EOs [16]. A typical method to test the efficacy of the volatile components of EOs is the inverted lid test that is based on the generation of EO vapor and the creation of an atmosphere at the selected temperature. This is not a standard assay although it is often recommended to evaluate the microbial inactivation by the volatile components of the EOs [17].

Based on the previously reported papers, it appears clear that it is very difficult to reliably compare data from the literature because many parameters have an influence on the results due to the strong variance of the used antimicrobial susceptibility testing methods, as reported by [18].

In the present work, in order to overcome safety risk during test execution, two Biosafety Level 1 bacteria, *S. epidermidis* and *E. coli*, were used. MIC values were first investigated for commercial thyme and oregano EOs against both bacteria by applying a microdilution test. After that, specific antimicrobial tests were set up to study the influence of main parameters (i.e., EO concentrations and absolute quantity, temperature of incubation, EO treatment duration considering time intervals larger than in previous studies) on EO efficacy in liquid and vapor phases; the obtained values were compared with MIC results. The results obtained with the selected bacteria were compared with available literature data on pathogenic strains to possibly validate them as surrogate microorganisms.

## 2. Results

### 2.1. Preliminary Test—Microdilution in Broth

The microdilution test was carried out diluting commercial thyme and oregano EOs with 1.5% TEGO; the EO concentrations were in the range 7.5–0.03 µg/mL. At the tested cultural conditions, only thyme EO inhibited the growth of *Staphylococcus epidermidis* and *Escherichia coli*, and the obtained MIC value was 7.5 µg/mL for both bacteria. On the contrary, for oregano EO, a MIC value was not obtained.

### 2.2. Agar Disk Diffusion Tests

In order to distinguish the activity of the EOs in the liquid and in the vapor phase, specific antimicrobial tests were set up against the chosen *S. epidermidis* and *E. coli* strains. As regard the liquid phase, the agar disk diffusion test was used to examine, at the same time, the EO effect in the liquid and vapor phase. As reported by [19], the inhibition zone caused by EOs depends on both the uniform diffusion of the substance into the agarized medium and the release of vapors above the tested bacteria. Generally, the antimicrobial efficacy of the EOs is evaluated at 24 h [20,21]; in the present work, in order to check the EO influence on the bacterial growth over time, inhibition was evaluated and compared at both 24 and 48 h.

#### 2.2.1. Test with a Gram-Positive Bacterium: *S. epidermidis*

**Figure 1:**
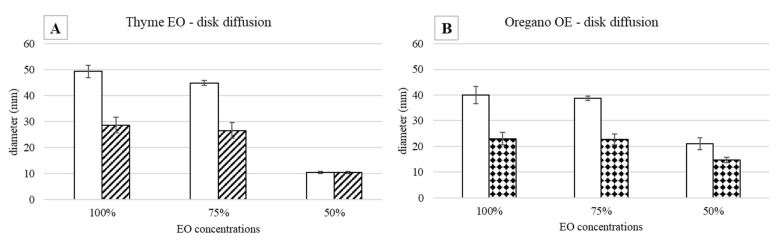
Agar disk diffusion test against *S. epidermidis* incubated at 37 °C: halo diameters measured after 24 (white bars) and 48 (drawn bars) h of incubation in the presence of (**A**) thyme and (**B**) oregano EOs.

The antimicrobial effect of 100%, 75%, and 50% EOs diluted with 1.5% TEGO on *S. epidermidis* grown at 37 °C is described. The measures of the inhibition halo diameter, checked after 24 and 48 h from the EO addition, are reported in Figure 1.

**Figure 2:**
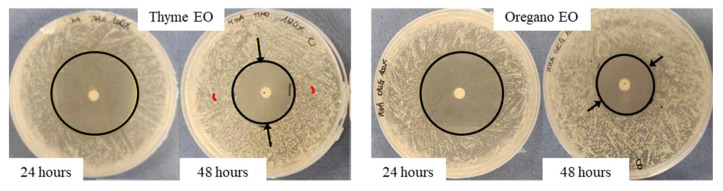
Variation of the inhibition halo diameter from 24 to 48 h, for the disk diffusion test, in the presence of 100% thyme or oregano EO, against *S. epidermidis*. Arrows indicate the variation direction.

As regards the thyme EO, it was able to influence bacterial growth at all the tested concentrations (Figure 1A). Looking at the changes of the halo diameters between 24 and 48 h of incubation, it is possible to observe that for the highest EO concentrations, the diameters decreased in a similar way, namely 41.9% for 100% EO and 40.9% for 75% EO, reaching, at the end of the incubation period comparable values, 28.6 mm and 26.5 mm respectively, which was not proportional to the EO concentration. On the contrary, in the presence of 50% EO, the diameter remained unchanged, i.e., equal to 10.4 mm, a value significantly lower with respect to that measured with the highest concentrations. Concerning the oregano EO, all the three concentrations allowed to measure inhibition halos, which varied between 24 and 48 h of incubation (Figure 1B). As observed for thyme EO, the effect due to 100% and 75% EO was very similar, and the respective diameters diminished by about the same percentage, namely 42.5% and 41.3%, reaching at 48 h 23 mm and 22.7 mm, respectively. In the case of oregano EO, for the 50% concentration, a decrease in halo diameter (30%) was also observed: at 48 h of incubation, the value was 14.7 mm. As an example, in Figure 2, it is possible to observe the decrease of the halo diameter in the presence of 100% thyme and oregano EOs.

#### 2.2.2. Test with a Gram-Negative Bacterium: *E. coli*

**Figure 3:**
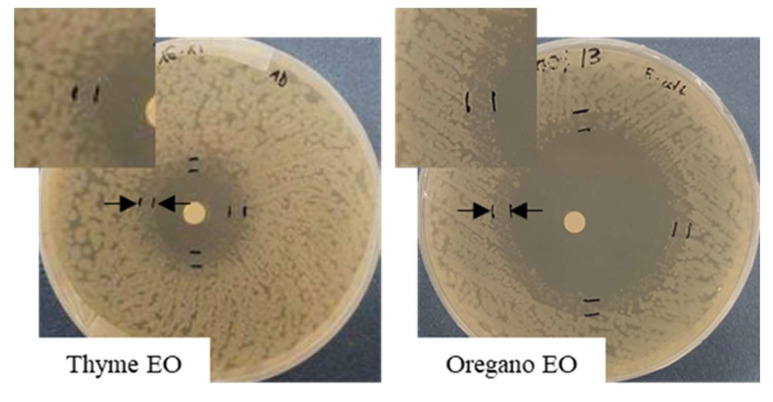
“Faint” microbial growth, shown between arrows and enlarged in the photo details, for *E. coli* growth in the presence of EOs in the disk diffusion assay.

The agar diffusion test was also carried out against *E. coli* grown at the optimal temperature of 28 °C. Differently from the results obtained in the presence of *S. epidermidis*, the haloes did not vary between 24 and 48 h of incubation; for this reason, only the results at 48 h are shown. Moreover, in all the tests performed in the presence of *E. coli*, a double halo was clearly visible, consisting in an inner portion, where the bacteria are absent, surrounded by a circular crown of “faint” microbial growth (Figure 3). According to the indications of [22] for the Enterobacteriaceae grown on Mueller–Hinton agar, the sum of the two inhibition haloes has to be considered as inhibition zone. In view of a possible dependence of the faint growth from the EO diffusion in the agar, the circular crown measures (mm) for each inhibition halo diameter are also reported.

**Figure 4:**
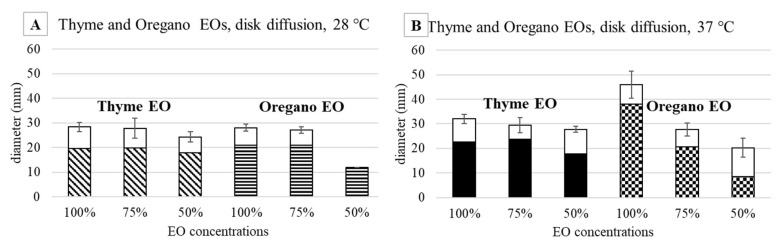
Agar diffusion test against *E. coli* incubated at 28 °C (**A**) and 37 °C (**B**): halo diameters measured after 48 h of incubation in the presence of thyme EO (oblique striped and black bars) and oregano EO (horizontal striped and checked bars). The white top portions of the bars represent the zones of faint bacterial growth.

Thyme EO, tested at 28 °C at 100 and 75%, allowed to obtain comparable results against *E. coli* (28.3 mm and 27.8 mm, respectively, Figure 4A). At the 50% concentration, the efficacy of thyme EO was lower, namely 24.3 mm, but comparable with respect to the higher concentrations. As regards the oregano EO, the efficacy at 100 and 75% was similar: 28 mm and 27 mm. At the same time, at 50%, the inhibition was less than half (12 mm).

The two EOs were also tested against *E. coli* grown at 37 °C (Figure 4B), the same incubation temperature of *S. epidermidis*, and the obtained results were compared with those at 28 °C. It is evident that at 37 °C, the inhibition was higher, independently from the EO used, in particular at 100% (32 mm and 46 mm for thyme and oregano, respectively).

### 2.3. Disk Volatilization Tests

The antibacterial properties of thyme and oregano volatile components were detected by means of disk volatilization test carried out in Petri dishes. In this case, the test was conducted with two different EO volumes, namely 3 and 10 µL, and paper disk diameters, namely 5 and 10 mm, respectively. The inhibition zone diameters were always measured at 24 and 48 h; in the tests with the higher EO volume, in the presence of *S. epidermidis*, the incubation was prolonged till 168 h, and the inhibition halo was evaluated each 24 h.

#### 2.3.1. Test with a Gram-Positive Bacterium: *S. epidermidis*

As previously reported for disk diffusion tests, also in this case, between 24 and 48 h of incubation, a progressive reduction of the inhibition zone was observed.

**Figure 5:**
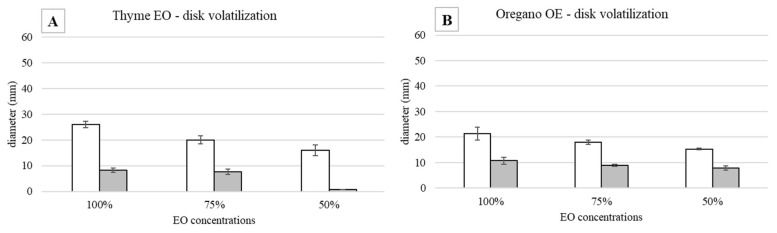
Disk volatilization test against *S. epidermidis* incubated at 37 °C: halo diameters measured after 24 (white bars) and 48 (grey bars) h of incubation in the presence of (**A**) thyme and (**B**) oregano EO (3 µL).

**Figure 6:**
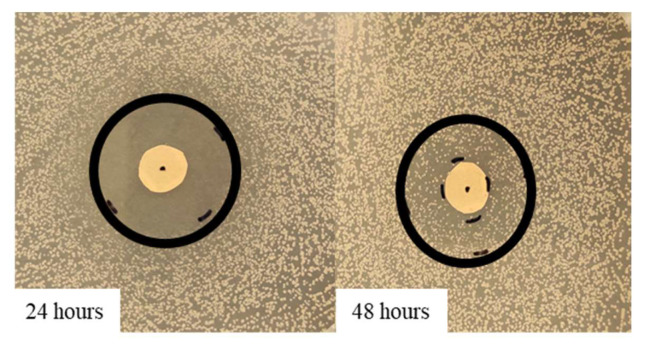
Variation of the inhibition halo diameter, from 24 to 48 h of incubation, for the disk volatilization test in the presence of 100% thyme EO against *S. epidermidis*.

As it is shown in Figure 5A,B, both EOs were able to influence *S. epidermidis* growth in a concentration-dependent way. In the first 24 h, in all the concentrations tested, the behavior of the inhibition halo was similar, with the measured diameters being equal to 26 mm, 20 mm, and 16 mm for thyme and 21.3 mm, 18 mm, and 15.3 mm for oregano EO, respectively. At 48 h, for both EOs, a bacterial growth recovery was observed, and the inhibition halo present at 24 h disappeared. In Figure 6, it is possible to identify two different zones: an outer portion with a normal bacterial growth and an inner one with a lower CFU number. For thyme EO, at 100% and 75%, the inner zones were 8.3 and 7.6 mm, corresponding, respectively, to 32 and 38%. For thyme at 50%, the bacterial growth completely covered the inhibition halo observed at 24 h. In the presence of oregano EO, the inner portions were equal to 50% in all the cases (10.7, 8.9, and 7.8 mm for 100, 75, and 50% EO, respectively).

The disk volatilization test conducted against *S. epidermidis* with 3 µL of EOs on 5 mm Ø paper disks (0.13 µL_EO_/mm^2^) gave a defined inhibition halo only in the first 24 h. To improve the inhibitory effect and to check how long it would be effective, the test was realized with 10 µL of 100, 75, and 50% EO on a 10 mm disk, maintaining a comparable ratio volume_EO_/paper disk area (0.15 µL_EO_/mm^2^), and the bacterial growth was prolonged for 168 h.

**Figure 7:**
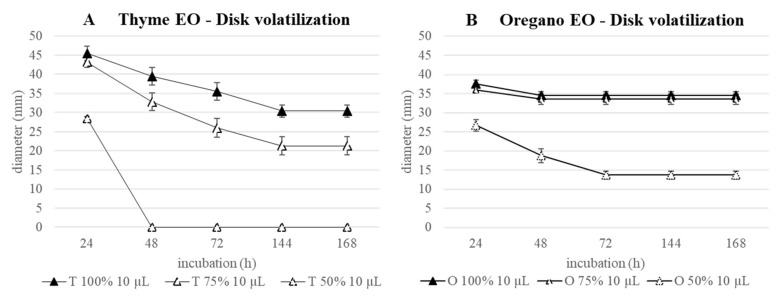
Disk volatilization test against *S. epidermidis*: results obtained with 10 µL of thyme (**A**) and oregano (**B**) EOs during 168 h of incubation.

As regards the thyme EO, at the two highest concentrations, a progressive halo reduction was measured until the 144th h, and then, stabilization of the inhibition was registered until the 168th hour (Figure 7A). At the end of the incubation, haloes were still high; in particular, that of 100% EO, i.e., 30.4 mm, was comparable to that obtained with 75% after 48 h (32.8 mm). Conversely, the 50% EO resulted as efficacious only in the first 24 h of treatment.

Considering 100 and 75% oregano EO, the curves were overlapped, with an equal slope between 24th and 48th h (Figure 7B). After that, the stabilization of the haloes was maintained until the end of the incubation, when 34.6 and 33.2 mm were measured. A different trend was obtained for EO 50%, characterized by a reduction of the inhibition halo till the 72nd h, followed by its stabilization. The final halo was 13.7 mm, which is 2.5 times lower than those measured with the highest EO concentrations.

The antimicrobial efficacy of oregano appears consistently superior to that of thyme at all the tested concentrations.

#### 2.3.2. Tests with a Gram-Negative Bacterium: *E. coli*

The antibacterial effect of the volatile phase of thyme and oregano EOs was also evaluated against *E. coli* grown at both 28 °C and 37 °C, utilizing 3 µL of EO on a paper disk of 5 mm Ø. Only 100% oregano EO was effective in the vapor phase; at 28 °C and 37 °C, comparable inhibition haloes (8.4 and 8.5 mm, respectively) were measured. The results obtained at 37 °C are reported in Table 1B and compared with those of *S. epidermidis*.

**Figure 8:**
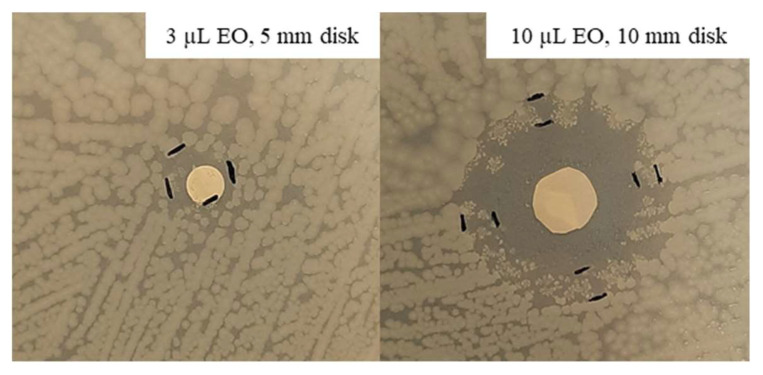
Disk volatilization test set-up with 100% oregano EO against *E. coli* after 48 h of incubation.

Considering the inhibition improvement obtained with a higher EO quantity (10 µL) against *S. epidermidis*, the same test was repeated against *E. coli* at 37 °C. After 48 h of incubation, an inhibition halo was measured for all the tested concentrations. The inhibition zones resulting from 100 and 75% thyme EO were similar, i.e., 38.5 and 35 mm, while that related to 50% was about halved, being equal to 19 mm. 100% Oregano EO allowed to obtain the highest inhibition halo, 43 mm, which is fivefold higher that that associated with 3 µL, while that related to 75% was slightly lower at 37.5 mm. Finally, 50% oregano EO showed an inhibition zone of 33 mm, comparable with those obtained with the highest concentrations of this EO. In Figure 8, an example of the obtained inhibition with 100% oregano EO, 3 and 10 µL, is shown.

As far as the agar disk diffusion assay is concerned, considering the results obtained at the 48th h of incubation at 37 °C, both the EOs show a better antibacterial effect against *E. coli*; indeed, the inhibition haloes are always higher than those measured for *S. epidermidis* (Table 1A). The greatest differences were evidenced in the application of 100% oregano EO and 50% thyme EO. In the first case, the inhibition zone related to *E. coli* was double than that of *S. epidermidis,* and in the second one, a halo about three-fold higher for *E. coli* was observed. For all the other EO concentrations, differences in the efficacy against the two bacteria were less remarkable and in the range 10–25%.

As regard the application of the disk volatilization assay (Table 1B), both thyme and oregano EOs at 3 and 10 µL showed the capability to inhibit the growth of *S. epidermidis* when applied in the vapor phase, with the only exception represented by 50% thyme EO, for which the inhibition zone was absent also for the higher EO volume. The antibacterial effect of 3 µL EOs against *E. coli* was null except for 100% oregano. The inhibition effect was greatly improved for all the concentrations of both EOs, increasing the volume. The inhibition due to 10 µL thyme EO against both the bacteria was comparable; on the contrary, that related to oregano was always higher against *E. coli*.

## 3. Discussion

Thyme and oregano EOs are well-known for their antimicrobial efficacy due to their carvacrol and thymol constituents [9,11]. In the present work, as it has been reported, the same MIC value was obtained with thyme EO against both the bacteria. On the contrary, at the tested concentrations, an MIC was not identified for oregano EO. In any case, both EOs were applied in the following specific antimicrobial tests, i.e., disk diffusion and disk volatilization, because the EO activity can vary depending on the application modality, namely in the liquid or in the vapor phase.

As regards the agar disk diffusion test, when it was applied against the Gram-positive bacterium, it showed a decrease of the inhibition halo diameter between 24 and 48 h of incubation; this behavior could be explained by the “threshold EO effect”; i.e., if the EO concentration is below this value, bacteria are allowed to grow [23]. On the contrary, this decrease was not evidenced for the Gram-negative strain, for which the inhibition was already stable after 24 h of incubation [24].

The disk volatilization test allowed to verify the antibacterial activity of the phenolic EO compounds due to the evaporation at 37 °C [25]. Both thyme and oregano EO were able to inhibit *S. epidermidis* growth in the first 24 h. After that, the inhibition halo was covered by a new bacterial growth, which was poor with respect to that on the rest of the agar surface, probably due to an EO concentration below the threshold value. The obtained results confirmed that the definition of the threshold concentration is of great importance because, when exposed to EO sub-lethal concentrations, bacteria can adapt to the presence of EOs in a similar manner to that of the antibiotic resistance, as previously reported by [26]. The antimicrobial properties of EO vapors depend, at the same time, on the relative volatility and the antimicrobial properties of their constituents [27,28]; the higher EO volume applied probably allowed to overcome the threshold value of carvacrol and thymol, the main active components of the two EOs [25]. Concerning *E. coli*, it showed a high resistance against the lowest volume (3 µL) of both EOs: in fact, only the maximum oregano EO concentration was inhibiting. When the higher EOs volume (10 µL) was applied, at all the concentrations, an inhibition was observed, probably in relation to an EO quantity above the threshold value.

Generally, in the literature, Gram-positive bacteria are reported to be more sensitive than Gram-negative ones regardless of the applied EO [29]; the results obtained in our work with thyme and oregano suggest that the antimicrobial properties of the EOs are mostly related to the individual susceptibility of the strain and change in relation to both the applied test (disk diffusion or disk volatilization) and EO concentration [24].

From the obtained results, it is clear that, with the EO volume being equal and with the paper disk diameter and the bacterial inoculum concentration, the inhibition obtained by both disk diffusion and disk volatilization methods were different. The detected efficacy after 48 h of incubation was always higher in the presence of a direct contact between the microorganism and the EO (the disk diffusion assays) independently of the tested oil and concentration.

In the case of the disk diffusion assay, the EO inhibitory effect is due to both the EO liquid phase in direct contact with the bacterial cells and the EO vapors inside the free head space; consequently, the haloes are higher than those obtained with the disk volatilization tests. Diameters measured at 48 h of incubation in the disk volatilization test are always lower than those obtained in the disk diffusion one. This observation could be explained by the fact that, in the last case, the measured inhibition halo was the result of the effect due to volatile compounds and those present in the liquid phase. Considering all the above observations, the application of both agar disk diffusion and disk volatilizations tests is mandatory.

The tests applied in the present work are the most frequently found in the literature due to their easy set-up and rapidity of results [29]. They are based on the principle of the Kirby–Bauer assay, which is usually utilized for antibiotic resistance studies [16]. The number of scientific reports about EO efficacy against clinical isolates, such as the Gram-positive *B. subtilis*, *S. aureus*, and *L. monocytogenes* and the Gram-negative *E. coli*, *S. typhimurium*, *P. aeruginosa*, and *Campylobacter* spp., is high in comparison with applications against non-pathogenic bacteria [29]. Taking in mind that the bacterial sensitivity to EOs depends on both the oil characteristics and the type of microorganism tested, it is clear that pathogenic clinical strains and non-pathogenic ones could differ in sensitivity against the same EO [30].

**Figure 9:**
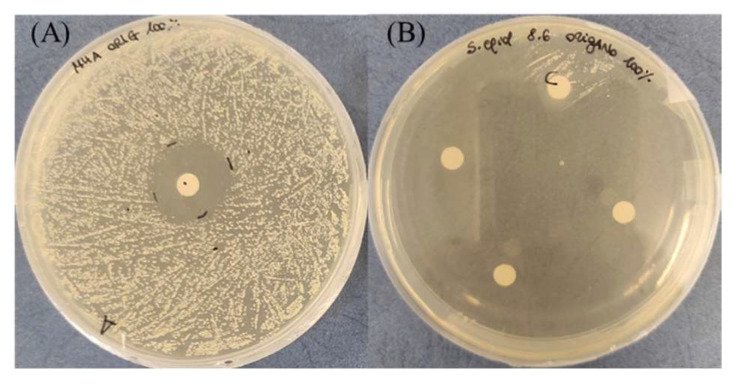
Inhibition effect due to a single (**A**) or three paper disks (**B**) impregnated with 100% oregano EO against *S. epidermidis*. In (**B**), a control disk with TEGO 1.5% (labeled “C”) was present.

The comparison between results obtained for pathogenic and non-pathogenic strains, although interesting, is not easy to be conducted; in fact, since the tested EO and the bacterial strain were equal, differences in the test set-up are represented by the EO volume, the paper disk diameter, and the CFU/mL of the inoculum. In analyzing the set-up, realized in recent times, from 2019 to 2021, it can be evidenced that microbial inoculum can vary from 10^5^ to 10^8^ CFU/mL [6,31,32,33,34,35,36]. The EO quantity can broadly differ from a minimum of 5 µL to a maximum increased up to four times [23,32,37,38]. Moreover, the CFU/mL and EO volume can be variously combined, bringing to different ratios “EO concentration in test area/CFU number”. This parameter is essential in the comparison of the EO efficacy. The number of EO-impregnated disks positioned in the Petri dish is another experimental condition that can vary: we demonstrated (see Figure 9) that is fundamental to put a single paper disk in each dish in order to avoid the additive effect dependent on the disk number; in most of the work, there may be three to five in each dish [23,37,38].

## 4. Materials and Methods

### 4.1. Microorganisms and Media

The tested standard strains, *Staphylococcus epidermidis* ATCC 12228 and *Escherichia coli* ATCC 8739, came from the Belgian Coordinated Collections of Micro-organisms (BCCM). They were maintained at +4 °C or cultivated at +37 °C on Mueller–Hinton agar (CM0337, Oxoid, Hampshire, UK) in aerobic conditions.

As indicated in the following chapters, in some antibacterial tests, *E. coli* was cultivated at 28 °C, using the same cultural medium. Mueller–Hinton broth (CM0405, Oxoid, Hampshire, UK) was utilized for the microdilution assays in well plates.

### 4.2. Essential Oils (EOs)

Commercial essential oils (EOs) from thyme (*Thymus vulgaris* EO, OE0970) and oregano (*Origanum vulgaris* leaf EO, OE0375) were purchased from Witt Italia Spa Company, Poirino, Italy. They were applied pure (100%) or diluted (75% *v*/*v* and 50% *v*/*v*) using a 1.5% solution of Polysorbate 20 (TEGO^®^ SML 20, Waltham, MA, USA). This solution was also used for the positive controls to check the potential interaction with bacterial growth.

### 4.3. Preliminary Test—Microdilution in Broth

The microdilution test was carried out in sterile, 96-well microplates according to CLSI standard method [35]. Thyme or oregano EOs were dissolved in sterile 1.5% TEGO to obtain a 15 µg/mL stock solution. Then, 100 µL of 2X Mueller–Hinton broth was poured in each well, while in the first column, 100 µL of the stock solution was added. Serial two-fold dilutions were directly prepared in the wells, obtaining the following concentrations: 7.5, 3.75, 1.875, 0.938, 0.469, 0.234, 0.117, 0.059, and 0.0285 µg/mL. A standardized inoculum was prepared in Mueller–Hinton broth to obtain, in each well, a final bacterial concentration equal to 5 × 10^5^ CFUmL^−1^. The total tested volume in each well was 200 µL. Biotic controls were set up with inoculated Mueller–Hinton broth together with un-inoculated abiotic controls for each tested concentration of EO; biotic and abiotic controls were also prepared with the cultural medium added with the 1.5% TEGO alone. Microplates were incubated at 37 °C in agitation at 150 rpm. After 24 h, MIC values were identified as the lowest EO concentrations that showed no bacterial growth as checked by the naked eye.

### 4.4. Antimicrobial Tests

**Figure 10:**
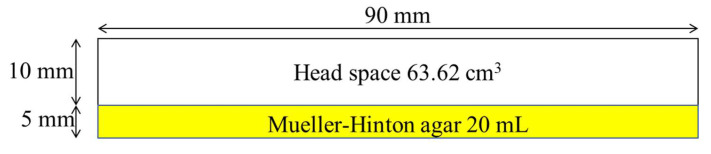
Dimensions of the Petri dishes filled with a fixed quantity of Mueller–Hinton agar, used for all the antibacterial tests.

In all the antibacterial tests, the inoculum was prepared using the direct colony suspension method. Three colonies, coming from a 24 h old culture grown on solid medium, were withdrawn and suspended into 5 mL of 0.85% NaCl to obtain a cell density equal to 1 × 10^8^ CFU/mL; this last density was further diluted, using the same saline solution, to reach the final concentration of 1 × 10^5^ CFU/mL. All the tests were performed in 90 mm Ø Petri dishes (Polystyrene, PS) filled with 20 mL of Mueller–Hinton agar, giving a 10 mm high head space corresponding to a free volume of 63.62 cm^3^ (Figure 10).

#### 4.4.1. Agar Disk Diffusion Test

The antimicrobial susceptibility testing for thyme and oregano EOs was carried out by applying the “Agar disk diffusion method” [39] using a single filter paper disc (Whatman N°1, 5 mm diameter) in each Petri dish. First, 100 µL suspensions of *S. epidermidis* or *E. coli*, 1.0 × 10^5^ CFU/mL, were spread over the surface of the Mueller–Hinton agar plates. One paper disc was gently placed in the center of each plate and then soaked with 3 µL of 100, 75, or 50% EO, or with 1.5% Polysorbate 20 (TEGO) for the control. Plates were sealed off with Parafilm^®^ (Neenah, WI, USA) and enveloped with PVC film to prevent EO vapor losses.

**Figure 11:**
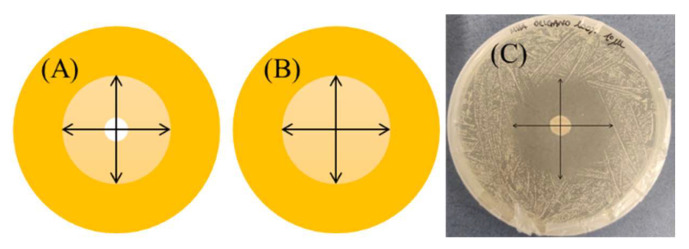
Inhibition zones measured (mm) at the two perpendicular diameters for the agar disk diffusion method (**A**) and the disk volatilization assay (**B**). (**C**) shows a real case measurement.

After 24 and 48 h of incubation, the inhibition zones at the two perpendicular diameters, as shown in Figure 11A, were measured by means of a ruler; the edges of the zones were read at the point of complete inhibition as judged by the naked eye. Each antibacterial test was performed in triplicate.

#### 4.4.2. Disk Volatilization Test

The disk volatilization assay was set up to test the antibacterial efficacy of the two EOs in vapor phase. To this purpose, a single filter paper disk was attached to the lid of the Petri dish by means of a bi-adhesive tape. Used disks had a diameter of 5 or 10 mm and were soaked, respectively, with 3 µL or 10 µL of 100, 75, or 50% EO or 1.5% TEGO. The obtained ratio “EO volume/disk area” were similar and, respectively, equal to 0.13 and 0.15 µL_EO_/mm^2^. An amount of 100 µL of 1.0 × 10^5^ CFU/mL bacterial suspension was spread over the surface of Mueller–Hinton agar. Once set up, Petri dishes sealed with Parafilm^®^ (Neenah, WI, USA) M and enveloped with PVC film were incubated inverted on top of the lid.

After 24 and 48 h of incubation, the inhibition zones on the agar surface below the imbibed paper disk were measured at the two perpendicular diameters at the point of complete inhibition as judged by the naked eye, Figure 11B. In some experiments, as indicated in the Results section, diameter measurements were carried out until the 168th h of incubation. Each trial was conducted in triplicate.

## 5. Conclusions

In the present work, *S. epidermidis* and *E. coli*, belonging to Biosafety Level 1, were chosen as indicators of the EO efficacy against a Gram-positive and a Gram-negative bacterium. The effect of commercial thyme and oregano EOs was tested by means of microdilution in broth, agar diffusion, and disk volatilization tests. As regards the first test, an equal MIC value was found against *S. epidermidis* and *E. coli* in the presence of thyme EO. Concerning the agar disk diffusion test, both bacteria were inhibited by all the tested EO concentrations. The inhibition obtained in the disk diffusion assays was higher than that in disk volatilization test; in the first case, the antimicrobial effect is due to both the EO components in the liquid and in the vapor phase.

The comparison of the obtained results with those present in the literature was not so easy; consequently, it can be concluded that when an assessment is needed, it is necessary to interpret the data with a high attention in relation to the applied analytical method used. In fact, even though standard methods are usually applied, numerous modifications are brought to adapt the set-up to the specific case study. Moreover, it is mandatory to evaluate the threshold value of the EOs concentration in the real conditions of application.

## Figures and Tables

**Table 1:** Comparison of the inhibition zones (mm) measured after 48 h of incubation at 37 °C in the presence of thyme or oregano EOs against *S. epidermidis* and *E. coli*. (**A**) Agar disk diffusion; (**B**) disk volatilization.

(A)	Thyme EO %			(B)	Thyme EO %		
	100	75	50		100	75	50
*S. epidermidis* 3 µL	28.6	26.5	10.4	*S. epidermidis* 3 µL	8.3	7.6	0
				10 µL	39.5	32.8	0
*E. coli* 3 µL	32.0	29.5	27.7	*E. coli* 3 µL	0	0	0
				10 µL	38.5	35	19
	**Oregano EO %**				**Oregano EO %**		
	**100**	**75**	**50**		**100**	**75**	**50**
*S. epidermidis* 3 µL	23.0	22.7	14.7	*S. epidermidis* 3 µL	10.7	8.9	7.8
				10 µL	34.6	33.5	18.0
*E. coli* 3 µL	46.0	27.7	20.3	*E. coli* 3 µL	8.5	0	0
				10 µL	43	37.5	33

## Data Availability

Not applicable.
